# One-Step ARMS-PCR for the Detection of SNPs—Using the Example of the *PADI4* Gene

**DOI:** 10.3390/mps2030063

**Published:** 2019-07-25

**Authors:** Sabrina Ehnert, Caren Linnemann, Bianca Braun, Josephine Botsch, Karolin Leibiger, Philipp Hemmann, Andreas K. Nussler

**Affiliations:** 1 Siegfried Weller Research Institute, Department of Trauma and Reconstructive Surgery, Eberhard Karls University Tübingen, BG Trauma Center Tübingen, 72076 Tübingen, Germany; 2 Interfakultäres Institut für Mikrobiologie und Infektionsmedizin Tübingen (IMIT), Institut für Medizinische Mikrobiologie und Hygiene, Eberhard Karls University Tübingen, 72076 Tübingen, Germany

**Keywords:** PAD4 (protein arginine deiminase 4), SNPs (single nucleotide polymorphisms), ARMS-PCR (tetra-primer amplification refractory mutation system PCR), PCR-RFLP (restriction fragment length polymorphism analysis), HRM-PCR (high resolution melting curve analysis)

## Abstract

In eukaryotes, cellular functions are tightly controlled by diverse post-translational modifications (PTMs) of proteins. One such PTM affecting many proteins is the deimination of arginine to citrulline. This process, called citrullination is catalyzed by a group of hydrolases called protein arginine deiminases (PADs), of which five isoforms have been identified. Hypercitrullination, as a result of increased PAD expression or activity, is associated with autoimmune diseases e.g., rheumatoid arthritis, lupus, Alzheimer’s disease, ulcerative colitis, multiple sclerosis, and certain cancers. Three common single nucleotide polymorphisms (SNPs) in the *PADI4* gene have been described, namely rs874881, rs11203366, and rs11203367, which are thought to affect PAD4 expression and activity. We here compared the suitability of four methods for the screening of SNPs in the *PADI4* gene: (i) SYBR-green based real-time polymerase chain reaction followed by high resolution melting curve analysis (HRM-PCR); (ii) PCR followed by detection of restriction fragment length polymorphisms (PCR-RFLP); (iii) conventional tetra-primer amplification refractory mutation system PCR (ARMS-PCR); and (iv) real-time PCR based on the one-step ARMS-PCR. Of these, ARMS-PCR proved to be the most suitable method regarding handling, duration, and cost of experiments. Using the method with SYBR-green based real-time PCR reagents further diminished handling steps and thus potential sources of error.

## 1. Introduction

In eukaryotes over 200 different post-translational modifications (PTMs) of proteins are described. Modifications of arginine are of particular interest, as arginine residues determine substrate specificity and control protein−protein and protein−DNA interactions. Arginine modifications include methylation, phosphorylation, ADP-ribosylation, and citrullination [1,2,3]. The latter is catalyzed by a small family of hydrolases called protein arginine deiminases (PADs). In human five PAD isoforms (PAD1–4 and PAD6) have been identified, encoded by five genes arrayed in tandem on chromosome 1 [2]. Of these, PADs 1−4 are catalytically active—the resulting deimination of arginine to citrulline results in a loss of the positive charge [2,4]. The tissue and substrate specificity of the PADs define their role in health and disease [5,6]: PAD1, which targets keratins, has been detected in epidermis, keratinocytes, sweat glands, and growing hair follicles. PAD2, targeting a broader spectrum of proteins, including histone H3, vimentin, enolase, and tubulin, is reported to be expressed in epidermis, keratinocytes, lymphocytes, monocytes, macrophages, neutrophils, oligodendrocytes, and Schwann cells. PAD3, which targets keratins and S100A3, is expressed in epidermis, keratinocytes, growing hair follicles, neutrophils, and Schwann cells. PAD4 being expressed in bone marrow, CD34^+^ cells, granulocytes, lymphocytes, monocytes, and macrophages, targets mainly histones H3 and H4, as well as the stress response gene OKL38 [7].

In particular, increased PAD4 expression and activity are associated with autoimmune diseases e.g., rheumatoid arthritis, lupus, Alzheimer’s disease, ulcerative colitis, multiple sclerosis, and certain cancers [8]. In this context it is of high interest to examine conditions that can alter PAD4 activity or expression levels. Among these there are single nucleotide polymorphisms (SNPs) of the *PADI4* gene. Three common SNPs of the *PADI4* gene, with a minor allele frequency (MAF) over 45%, are described for human, for overview see Table 1. These SNPs are thought to stabilize *PADI4* mRNA and thus affect PAD4 levels and activity [9]. For rheumatoid arthritis a positive correlation between SNPs of the *PADI4* gene and the susceptibility for and severity of the disease has been described [10].

To prove that SNPs of the *PADI4* gene correlate with other diseases or health conditions, a large amount of patients needs to be screened. Due to high costs this cannot be done routinely by sequencing, but requires a robust, accurate, easy to use, and cost effective method for the detection of these SNPs. Therefore, we compared three commonly used methods for the detection of the three SNPs of the *PADI4* gene (rs874881, rs11203366, and rs11203367): (i) SYBR-green based real-time polymerase chain reaction followed by high resolution melting curves (HRM-PCR); (ii) PCR followed by restriction fragment length polymorphism (PCR-RFLP); and (iii) tetra-primer amplification refractory mutation system PCR (ARMS-PCR) [11,12]. Of these, ARMS-PCR proved to be the most suitable. Thus, the method was optimized to further reduce handling steps and costs. The methods were summarized to provide an overview on handling and possible pitfalls, such that the method can be easily applied to detect other SNPs.

## 2. Materials and Methods

### 2.1. Individuals

Swabs of the mucous membrane were collected from 35 healthy volunteers: 18 male and 17 female donors. Each subject was informed about the study and the signed consent was obtained (ethical vote 756/2018BO2 approved 24.09.2018).

### 2.2. DNA Extraction

**Figure 1:**
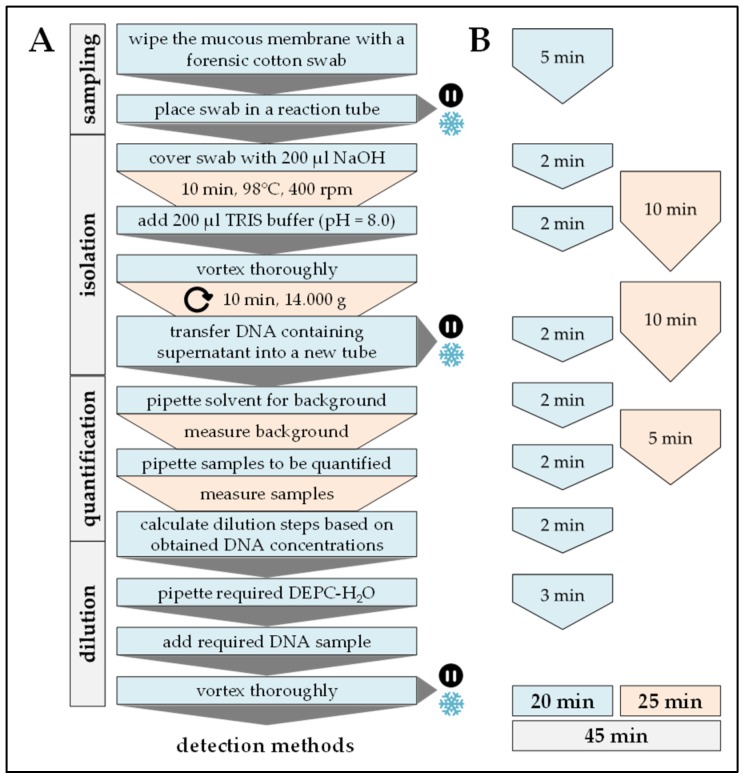
Workflow and timing of DNA sampling and isolation. In (**A**) workflow and (**B**) timing the blue color marks handling steps (time) of the experimenter and the orange color marks pause times caused by the procedure. 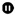 Procedure can be stopped at this step. 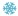 Store samples at −20 °C/prevents repetitive freeze and thaw cycles.

Swabs of the mucous membrane were obtained using DNA and DNase free forensic cotton swabs (Sarstedt, Nümbrecht, Germany). Cotton swabs were transferred into a sterile reaction tube and covered with 20 µL of 50 mM NaOH. After 10 min incubation at 98 °C with gentle agitation (250 rpm) 200 µL of a 100 mM Tris buffer (pH = 8.0) was added to neutralize the pH. Insoluble fragments were removed by centrifugation (10 min at 14,000× *g*). Resulting DNA samples were quantified photometrically with a FLUOstar Omega plate reader (BMG Labtech, Ortenberg, Germany) with the LVis Plate (BMG Labtech). Using this method samples should have a DNA concentration between 50 and 200 ng/µL. For further experiments DNA samples are diluted to a concentration of 50 ng/µL with DEPC-H_2_O. DNA samples can be stored at −20 °C for at least two months (but: Prevent repetitive freeze and thaw cycles). Workflow and timing are summarized in Figure 1.

### 2.3. Primer Information

Primers suitable for all three methods were designed using a program developed by Ye (http://primer1.soton.ac.uk/primer1.html) [13]. The target DNA sequences were obtained from the Ensembl databank (ENSG00000159339). Primers were obtained from Eurofins Genomics (Ebersberg, Germany). Primer sequences and binding sites are listed in Table 2.

### 2.4. HRM-PCR

High resolution melting (HRM) analysis of SYBR-green based PCR products was performed with a StepOnePlus Real-Time PCR System (Applied Biosystems, Foster City, CA, USA). Base for the PCR was the GreenMasterMix (2x) High ROX (Genaxxon, Ulm, Germany). For this method only the outer primers (outer forward primer—OFP and outer reverse primer—ORP/Table 2) were used, all in a 1:1 ratio. Thus, the final reaction volume of 20 µL contained 100 ng of genomic DNA, and 1 pmol of each primer. The PCRs were all run with an annealing temperature of 65 °C. After 40 cycles of amplification, a melting curve was plotted with a starting temperature of 75 °C and increasing steps of 0.1 °C up to 90 °C [14]. Resulting melting curves were exported from the StepOne software Version 3.0 (Applied Biosystems, Foster City, CA, USA) for further analysis with MS excel and GraphPad Prism (Version 5, El Camino Real, CA USA).

**Figure 2:**
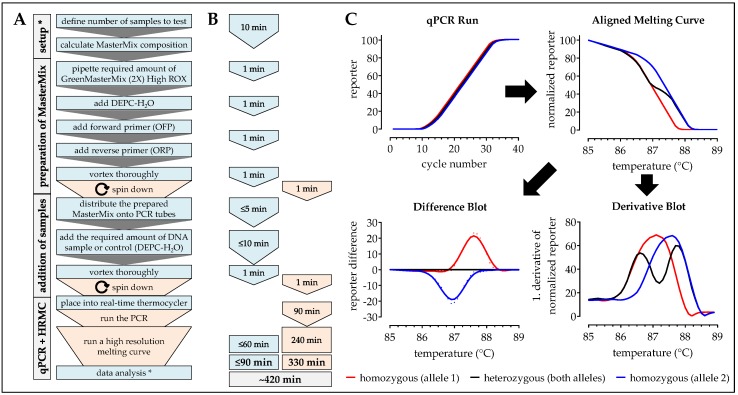
Workflow, timing, and expected results for the analysis of SNPs using the high resolution melting curves (HRM)-PCR method. In (**A**) workflow and (**B**) timing the blue color marks handling steps (time) of the experimenter and the orange color marks pause times caused by the procedure. (**C**) Expected results: While the PCR is running fluorescent (SYBR green) signal should increase comparably between all samples. The following high resolution melting analysis includes an aligned melting curve, as well as the resulting difference blot (difference of the normalized reporter with heterozygous samples as reference) and derivative blot (1. derivative of the normalized reporter). * When the method is frequently done a calculation matrix should be used.

Aligned Melting Curves (fluorescence intensities (f.i.) of the reporter (SYBR-green) were calculated in a two-step procedure: In the first step the curve was shifted vertically so that the smallest value equals zero (each reporter value (f.i.) – minimum reporter value (f.i.)); in the second step the maximum intensity of the reporter was equalized (each reporter value (f.i.) * 100/maximum reporter value (f.i.)). For the difference blot the mean of the heterozygous samples (normalized reporter) was set as reference. Each normalized reporter value was subtracted from this reference. The derivative blot was generated using the derivative function in GraphPad Prism (smoothened by 4th order and six neighbors on each size). Workflow and expected results are summarized in Figure 2.

### 2.5. PCR-RFLP

**Figure 3:**
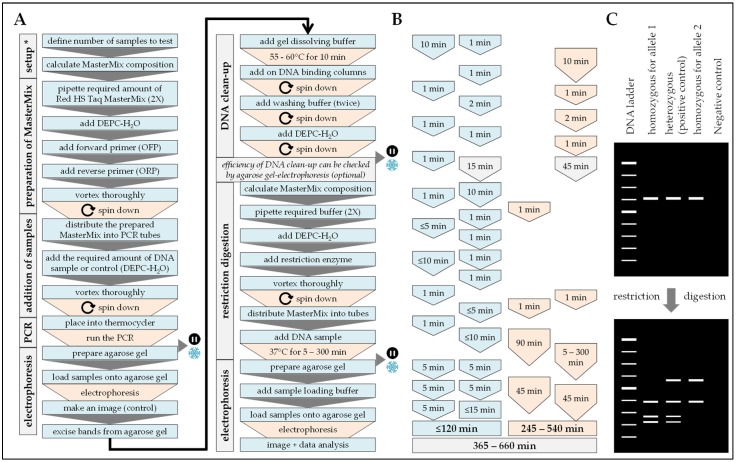
Workflow, timing, and expected results for the analysis of SNPs using the PCR- restriction fragment length polymorphisms (RFLP) method. In (**A**) workflow and (**B**) timing the blue color marks handling steps (time) of the experimenter and the orange color marks pause times caused by the procedure. (**C**) Expected results: The PCR products should have the size defined by the two outer primers (OFP and ORP). After the restriction digestion of the purified PCR products, the band pattern should be characteristic for homozygous or heterozygous samples. Possible restriction enzymes and the expected cutting pattern to detect the *PADI4* SNPs are summarized in supplementary Figure S1. * When the method is frequently done a calculation matrix should be used. 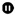 Procedure can be stopped at this step. 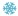 Store samples at −20 °C/prevent repetitive freeze and thaw cycles.

PCR-RFLP analysis begins with a conventional PCR performed in an Arktik Thermal Cycler (ThermoScientific, Waltham, MA, USA). Base for the PCR was the Red HS Taq Master Mix (2X/Biozym, Hessisch Oldendorf, Germany), which was used according to the manufacturers instructions. Similar to the HRM-PCR only the outer primers (OFP and ORP/Table 2) were used, all in a 1:1 ratio, with an annealing temperature of 65 °C for all primers. Here the final reaction volume of 20 µL contained 200 ng of genomic DNA, and 10 pmol of each outer primer (OFP and ORP). Resulting PCR products were separated from the genomic DNA by agarose gel electrophoresis (1.5% agarose gel) and visualized with ethidium bromide. Amplicon bands were isolated from the agarose gel using the ReliaPrepTM DNA Clean-up and Concentration System (Promega, Madison, WI, USA) to obtain clear results in the following DNA digestion. The purified DNA was digested with restriction enzymes leading to mutation specific DNA fragments—all possible restriction enzymes and corresponding restriction pattern were identified with the help of the NEBcutter: http://www.labtools.us/nebcutter-v2-0/ and SNP cutter http://bioinfo.bsd.uchicago.edu/SNP_cutter [15] (supplementary Figure S1). Here, the restriction enzymes Sau96I (for SNP 163), SsiI also known as AciI (for SNP 245), and HpaII (for SNP 335) were used. The resulting fragments were again separated by agarose gel electrophoresis (2% agarose gel) and visualized with ethidium bromide. Workflow and expected results are summarized in Figure 3.

### 2.6. ARMS-PCR

For the SNP characterization with ARMS-PCR, all four primers designed for each SNP (Table 2) were used. While the outer (flanking) primers served as an internal control for the integrity of the DNA, the two inner primers target DNA fragments with different lengths, which were specific for the two alleles to be compared (Table 2). The PCR was run in the Arktik Thermal Cycler using the Red HS Taq Master Mix (2X) as described above. A PCR reaction volume of 10 µL was sufficient for clear results. The amount of genomic DNA and primers, as well as the annealing temperature were optimized for each SNP (Table 3) [11,16]. Conventional PCR started with an initial denaturation at 95 °C for 2 min, followed by 40 cycles of denaturation (95 °C, 15 s), annealing (primer specific T_a_ see Table 3, 15 s), and elongation (72 °C, 15 s). The reaction was terminated by a final elongation step of 72 °C for 10 min. PCR conditions were optimized for the used Red HS Taq Master Mix (2X).

**Figure 4:**
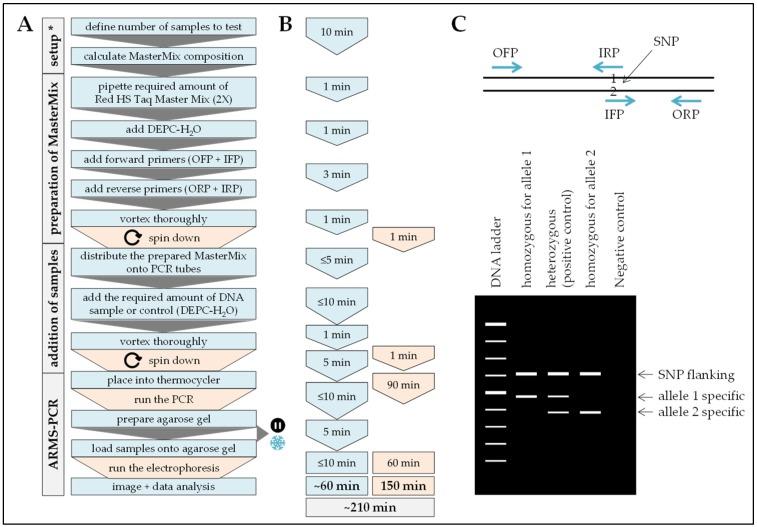
Workflow, timing, and expected results for the analysis of SNPs using the ARMS-PCR method. In (**A**) workflow and (**B**) timing the blue color marks handling steps (time) of the experimenter and the orange color marks pause times caused by the procedure. (**C**) Expected results: All PCR products should have the largest size band defined by the two outer primers (OFP and ORP). Homozygous samples should show only one additional band—their length is defined by the allele specific inner primers. Heterozygous samples should show both additional bands. * When the method is frequently done a calculation matrix should be used. 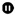 Procedure can be stopped at this step. 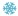 Store samples at −20 °C/prevents repetitive freeze and thaw cycles.

The amplified DNA was separated by electrophoresis using a 2% agarose gel. DNA bands were visualized with ethidium bromide and a GelDoc (Intas Science Imaging Instruments, Göttingen, Germany). The contrast of the images was adjusted and the bands colored in a fire spectrum by ImageJ Fiji (grayscale image → image → lookup tables → fire) for better interpretation of the bands. Workflow and expected results are summarized in Figure 4.

### 2.7. One-Step ARMS-PCR

**Figure 5:**
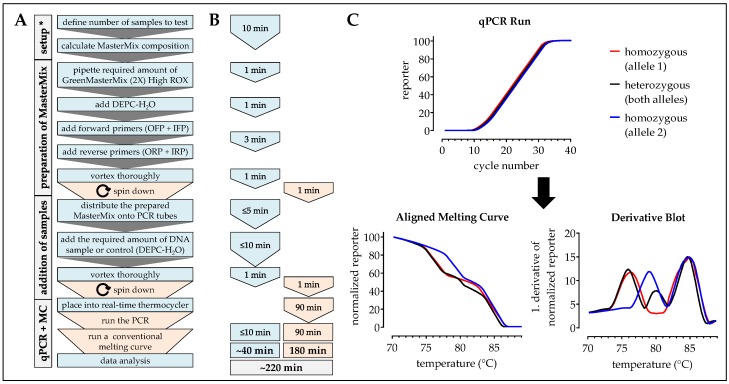
Workflow, timing, and expected results for the analysis of SNPs using the modified one-step ARMS-PCR method. In (**A**) workflow and (**B**) timing the blue color marks handling steps (time) of the experimenter and the orange color marks pause times caused by the procedure. (**C**) Expected results: While the PCR is running a fluorescent (SYBR green) signal should increase comparably between all samples. The following melting curve analysis (aligned melting curve and derivative blot) can be done without additional calculation steps. * When the method is frequently done a calculation matrix should be used.

In order to reduce time and handling steps ARMS-PCR method was modified as a one-step procedure by using SYBR-green based PCR GreenMasterMix (2x) High ROX (Genaxxon) with a StepOnePlus Real-Time PCR System (Applied Biosystems), as described above. Total volume of the ARMS-PCR reaction mixture was 20 µL. The amount of DNA template and primers was adjusted to the volume (Table 3). The PCRs were all run with an annealing temperature of 65 °C. After 40 cycles of amplification, a melting curve was plotted with a starting temperature of 70 °C and increasing steps of 0.3 °C up to 90 °C. Resulting melting curves were examined using the StepOne software Version 3.0 (Applied Biosystems, Foster City, CA, USA). For visualization data were exported to GraphPad Prism (Version 5, El Camino Real, CA, USA). Workflow and expected results are summarized in Figure 5.

## 3. Results

### 3.1. Comparison of the Different Methods Regarding Timing, Handling, and Costs

HRM-PCR, PCR-RFLP, and ARMS-PCR were initially considered as these methods are frequently used to detect SNPs. Time required for the DNA isolation was identical for all tested methods (~20 min/35 samples). Time required for pipetting was lowest for HRM-PCR (~30 min/35 samples) and ARMS-PCR (~50 min/35 samples). Most pipetting time was needed for PCR-RFLP (~105 min/35 samples). Pause times caused by the procedure were shortest for ARMS-PCR (~150 min). Pause times for HRM-PCR (~330 min) and PCR-RFLP (~320 min) were comparable. Final analysis of the result was fastest for ARMS-PCR (~10 min/35 samples) and PCR-RFLP (~15 min/35 samples) due to fast comparison of band patterns. Analysis of the data from HRM-PCR required additional calculation steps, to obtain an aligned melting curve, as well as a difference blot and a derivative blot—the informative value of these graphs varies between the SNPs. Time for the additional calculations can be limited by using a calculation matrix (~60 min/35 samples). In order to reduce handling steps ARMS-PCR method was modified as a one-step procedure: Time required for pipetting was reduced to ~30 min/35 samples for an increased pause time caused by the procedure. Interpretation of the results (~10 min/35 samples) was done with the StepOne software and did not require additional calculation steps. Depending on the base-change of the SNPs informative value and ease of interpretation differed between the methods. Experimental costs for the different detection methods are summarized in Table 4.

### 3.2. Interpretation of the Results with the Four Different Methods

#### 3.2.1. SNP 163A>G

**Figure 6:**
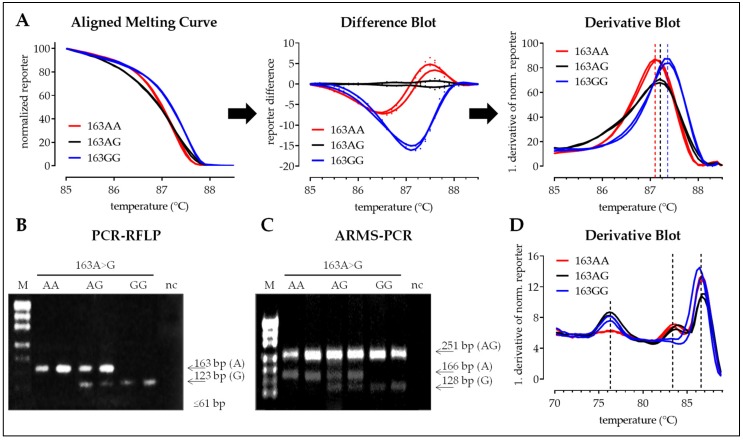
Detection of SNP 163A>G with four different methods: HRM-PCR, PCR-RFLP, conventional, and one-step ARMS-PCR. (**A**) High resolution melting curve analysis following SNP flanking PCR can be done using the aligned melting curve (normalized reporter), the resulting difference blot (difference of the normalized reporter with heterozygous samples as reference), or derivative blot (1. derivative of the normalized reporter). (**B**) Representative band pattern following enzymatic digestion of the SNP flanking PCR products with Sau96I (PCR-RFLP). (**C**) Representative band pattern of the conventional ARMS-PCR. (**D**) Melting curve analysis following one-step ARMS-PCR is performed with the derivative blot (1. derivative of the reporter). Presented data for the three methods were obtained from the same six samples.

For HRM analysis of PCR products for SNP 163A>G an aligned melting curve, a difference blot and a derivative blot were generated (Figure 6A). The aligned melting curve with the normalized reporter showed only small differences between the different alleles. The derivative blot had slightly higher informative value, showing a clear shift in the curves between the two homozygous samples (163AA and 163GG), with a lower melting temperature for the A-alleles. Compared to that curves for heterozygous samples (163AG) were more flat. However, for SNP 163A>G the best interpretation of HRM-pPCR data was given by the difference blot (Figure 6A). In contrast band pattern of the PCR-RFLP and the ARMS-PCR were easy to interpret, with weaker band intensities for PCR-RFLP (Figure 6B,C). In case of the modified one-step ARMS-PCR a simple derivative blot showing the 1. derivative of the reporter was sufficient for interpretation. Depending on the sample the derivative blot showed two (homozygous samples) or three (heterozygous samples) peaks with separated melting temperatures (Figure 6D).

#### 3.2.2. SNP 245C>T

**Figure 7:**
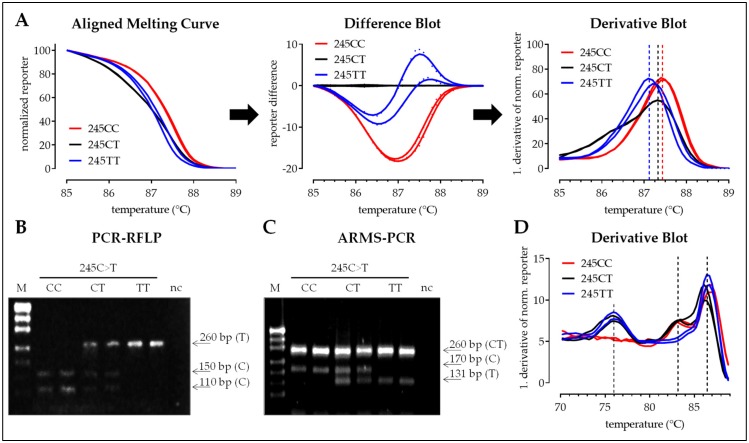
Detection of SNP 245C>T with four different methods: HRM-PCR, PCR-RFLP, conventional, and one-step ARMS-PCR. (**A**) High resolution melting curve analysis following SNP flanking PCR can be done using the aligned melting curve (normalized reporter), the resulting difference blot (difference of the normalized reporter with heterozygous samples as reference), or derivative blot (1. derivative of the normalized reporter). (**B**) Representative band pattern following enzymatic digestion of the SNP flanking PCR products with SsiI also known as AciI (PCR-RFLP). (**C**) Representative band pattern of the conventional ARMS-PCR. (**D**) Melting curve analysis following one-step ARMS-PCR is performed with the derivative blot (1. derivative of the reporter). Presented data for the three methods were obtained from the same six samples.

For detection of SNP 245C>T by HRM-PCR again an aligned melting curve, a difference blot and a derivative blot were generated. Similar to SNP 163A>G the aligned melting curve showed only small differences between the different alleles. Differences in the derivative blot showed a clear shift in the curves between the two homozygous samples (245CC and 245TT), with a lower melting temperature for the T-alleles. Compared to the curves of the homozygous samples, curves for heterozygous samples (245CT) were more flat. As for SNP 163A>G best interpretation of HRM-PCR data for SNP 245C>T was given by the difference blot (Figure 7A). For SNP 245C>T the band pattern of the PCR-RFLP and the ARMS-PCR were also easy to interpret, although the intensity of bands was weaker for PCR-RFLP (Figure 7B,C). The modified one-step ARMS-PCR was analyzed using a simple derivative blot, showing two (homozygous samples) or three (heterozygous samples) peaks with separated melting temperatures (Figure 7D).

#### 3.2.3. SNP 335C>G

**Figure 8:**
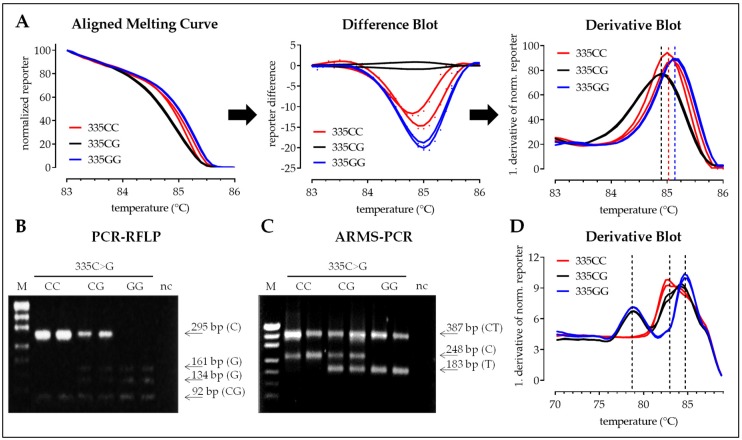
Detection of SNP 335C>G with four different methods: HRM-PCR, PCR-RFLP, conventional, and one-step ARMS-PCR. (**A**) High resolution melting curve analysis following SNP flanking PCR can be done using the aligned melting curve (normalized reporter), the resulting difference blot (difference of the normalized reporter with heterozygous samples as reference), or derivative blot (1. derivative of the normalized reporter). (**B**) Representative band pattern following enzymatic digestion of the SNP flanking PCR products with HpaII (PCR-RFLP). (**C**) Representative band pattern of the conventional ARMS-PCR. (**D**) Melting curve analysis following one-step ARMS-PCR is performed with the derivative blot (1. derivative of the reporter). Presented data for the three methods were obtained from the same six samples.

Compared to the other SNPs, differences in the aligned melting curves (HRM-PCR) were lower for SNP 335C>G. Similarly, differences between curves of the two homozygous samples (335CC and 335GG) were hard to detect in the derivative blot, with a melting temperature for the C-alleles slightly lower than for the G-alleles. Curves for heterozygous samples (335CG) could be differentiated by their flat shape. In contrast to the other SNPs investigated, the difference blot gave no clear result for SNP 335C>G (Figure 8A). For this SNP band pattern of the PCR-RFLP and the ARMS-PCR were much easier to interpret than HRM-PCR data, with weaker band intensities for PCR-RFLP (Figure 8B,C). For the interpretation of the modified one-step ARMS-PCR a simple derivative blot was sufficient. Peaks for the G-allele specific products were clearly separated from the other two peaks. Peaks for the C-allele specific products and the SNP flanking products were located close to each other, resulting in one wide peak (Figure 8D).

### 3.3. SNP Distribution Within the Samples

35 voluntary donors were screened for the three SNPs in the *PADI4* gene. In case of SNP 163A>G seven donors (four female and three male/20.0%) were homozygous for the major allele (A-allele) and eight donors (one female and seven male/22.9%) were homozygous for the minor allele (G-allele). The remaining 20 donors (12 female and eight male/57.1%) were heterozygous for SNP 163A>G. Regarding SNP 245C>T eight donors (four female and four male/22.9%) were homozygous for the major allele (C-allele) and seven donors (one female and six male/20.0%) were homozygous for the minor allele (T-allele). 20 donors (12 female and eight male/57.1%) were heterozygous for SNP 245C>T. Similarly, eight donors (four female and four male/22.9%) were homozygous for the major allele (C-allele) and seven donors (one female and six male/20.0%) were homozygous for the minor allele (G-allele) of SNP 335C>G. The remaining 20 donors (12 female and eight male/57.1%) were heterozygous for SNP 335C>G.

Interestingly, there was a high correlation between the SNPs. Seven donors were homozygous for the minor alleles and six donors were homozygous for the major alleles of all three SNPs of the *PADI4* gene. Similarly, 18 donors were heterozygous for all three SNPs of the *PADI4* gene. Only four donors were homozygous for one or two of the investigated SNPS and heterozygous for the other(s).

## 4. Discussion

As shown over the past years, SNPs can have an impact on susceptibility, development, and severity of many diseases [17]. To characterize SNP associated phenotypes, it is necessary to have a suitable screening method for SNPs. We first compared three conventional SNP detection methods in our study, namely HRM-PCR, PCR-RFLP, and ARMS-PCR, to detect three common SNPs of the *PADI4* gene. These *PADI4* SNPs are associated with diseases e.g., rheumatoid arthritis, lupus, Alzheimer’s disease, ulcerative colitis, multiple sclerosis, and certain cancers [8,10].

Although HRM-PCR has the least handling steps and thus the lowest risk for individual mistakes, interpretation of results was a lot easier and faster with the other two methods. HRM-PCR required additional calculation steps to successfully interpret the data—although, these calculations can be done with MS excel and GraphPad Prism investing into specific analysis software could be beneficial to reduce time and effort for analysis. The ease of interpretation is also strongly dependent on the SNP to be detected: Base inclusion or deletion resulting in a frameshift usually result in a bigger change in melting temperature than base-exchanges as in our three SNPs—here the actual exchange affects the melting temperature. For example, data interpretation for SNPs 163A>G and 245C>T was more distinct than for SNP 335C>G. This can be explained by the resulting change in the number of hydrogen bonds when adenine (A) or thymine (T) are replaced by guanine (G) or cytosine (C). The numbers of hydrogen bonds, which primarily define the melting temperature, do not change with an A↔T or C↔G exchange [14]. This is the case for SNP 335C>G, where the change in melting temperature solely depends on the tertiary structure of the amplicon and is thus hard to detect. These points have to be considered when the HRM-PCR method shall be used to detect SNPs. Due to the necessity to detect small changes it is advisable to include a heterozygous sample as well as the two homozygous samples as positive controls for each run.

Interpretation of results with the PCR-RFLP method was comparable to the ARMS-PCR method, although band intensities were weaker for the PCR-RFLP method. Noteworthy, an incomplete restriction digestion can lead to a wrong interpretation of the results. Similar to HRM-PCR a heterozygous sample as well as the two homozygous samples should be included as positive controls for each run. Biggest drawback for this method was that it required a lot more handling steps (possible sources of errors) and time. Material costs were also a lot higher for PCR-RFLP. PCR-RFLP method has the advantage that it can be performed with standard laboratory materials and equipment. However, PCR-RFLP requires most time and handling steps of the method tested, and is therefore considered as most expensive and most prone to errors—this favors the use of the other methods. PCR-RFLP is a classic method of genotyping that is based on endonuclease cleavage thus has to be considered that this method is not suitable for all SNPs, as the method is limited to SNPs resulting in a change in known restriction sites [15,18].

Overall ARMS-PCR was the most beneficial method. Although, running the electrophoresis required additional handling steps (source of errors) compared to the HRM-PCR method, interpretation of the results was much easier and faster. The only disadvantage of ARMS-PCR is the optimization of the PCR with the four primers. That might be a laborious process dependent on the primer design [16]. The work of Mesrian Tanha and colleagues suggests to include an additional mismatch at −2 positions of the outer primers and to equalize annealing temperatures during primer design in order to improve the genotyping procedure with the ARMS-PCR method [19]. Using only a heterozygous sample as positive control is sufficient due to the simple band pattern to be expected. Besides the easy and effortless interpretation of the ARMS-PCR results, this method has the advantage that it can be performed with standard laboratory materials and equipment. Therefore, it is a very good choice to screen for possible pathophysiological roles of SNPs.

For high-throughput screening of samples it is advisable to minimize handling steps in order to reduce sources of errors. Thus, real-time PCR based methods, e.g., the HRM-PCR is of advantage. In order to reduce time and pipetting steps with the so far favored ARMS-PCR method we modified the procedure into a one-step procedure using SYBR-green based real-time PCR technology. By using all four ARMS-PCR primers the size of the expected amplicons differs significantly. Thus, a conventional and fast melting curve was sufficient to identify the specific peaks. Furthermore, no additional calculation steps or software was needed to interpret the results, which are independent on the gel detection method. Similar to the conventional ARMS-PCR only a heterozygous sample is sufficient as positive control.

**Figure 9:**
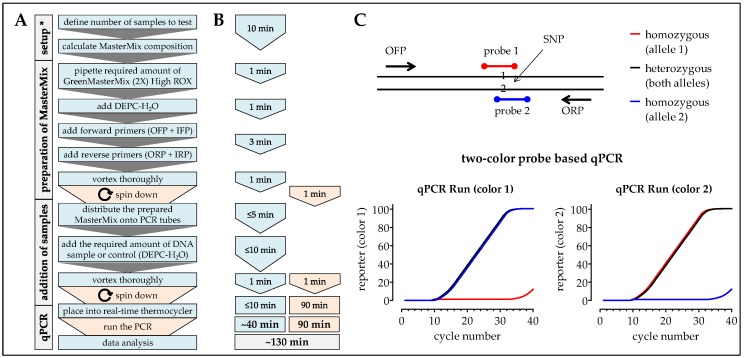
Modification of the ARMS-PCR method using probe based real-time PCR technology—proposed workflow, timing, and expected results. In (**A**) workflow and (**B**) timing the blue color marks handling steps (time) of the experimenter and the orange color marks pause times caused by the procedure. (**C**) Expected results: While the PCR is running a fluorescent signal for both used reporters is detected online. For heterozygous samples both reporters will increase with time. For homozygous samples only one of the reporters will increase with time, depending on the allele present. * When the method is frequently done a calculation matrix should be used.

If time should be further reduced, it is conceivable to modify the optimized ARMS-PCR protocol for using probe based real-time PCR technology. Therefore, the inner primer sequences have to be labeled with two different fluorescent reporters at the 5′-end (for example FAM; i.e., 6-carboxyfluorescein) and a corresponding quencher at the 3′-end (for example TAMRA; i.e., 6-carboxy-tetramethylrhodamine) [20] in order to use the 5′ nuclease activity of Taq polymerase first described by Holland et al. [21]. The nuclease degradation of the hybridization probe releases the reporter during the extension phase of PCR, resulting in an increase in reporter fluorescence. Using real-time PCR equipment, the data can be interpreted easily online (one or two reporters detected) without the need for a time-consuming melting curve and its analysis. Workflow and expected results are summarized in Figure 9. If no suitable real-time PCR equipment is available, the PCR can be performed with a classical thermocycler and formed fluorescence can be detected with a micro-plate reader similar to the method described using energy-transfer-labeled primers [22,23]. The additional material costs for the probes will be compensated by the easy and fast online interpretation of the data. With the minimized handling steps and the reduced time the proposed method is ideal for high-throughput screenings.

Overall, screening our 35 samples revealed good comparability of the tested methods. ARMS-PCR was the most beneficial, as it can be used with standard laboratory equipment. The handling steps were manageable and interpretation of results was easy and fast. Interestingly, there was a high correlation between the three screened SNPs. Samples homozygous for one minor (major) allele were frequently also homozygous for the other two minor (major) alleles. Considering that all three SNPs code for a missense mutation (amino-acid exchange), the resulting proteins differ in three amino acids located at the N-terminal end—raising the question, if there is any advantage in possessing all three mutations, so that they would evolve together. The impact of these changes is still unknown and needs to be examined. So far no effect of the single SNPs on the enzymatic activity has been described, but a stabilizing effect on the mRNA of *PADI4* has been proposed [10]. That would result in a higher number of active proteins and thus in an increased citrullination capacity.

## 5. Conclusions

In summary, HRM-PCR had the advantage of the least handling steps and thus least sources of errors, however, interpretation of the results required additional calculation steps and was not as easy and unambiguous as for the other two methods. Although ARMS-PCR requires more handling steps than HRM-PCR it was the most cost- and time-effective method tried, due to the easy and fast interpretation of the results, with the advantage that the method can be optimized for any SNP and can be performed with standard laboratory equipment. Although interpretation of results was comparably easy using the PCR-RFLP methods, a lot more handling steps, longer pause times, and material costs disqualified this method. Modification of the ARMS-PCR method into a one-step procedure using real-time PCR technology could be an option to reduce handling steps while simplifying the interpretation of the results.

## Figures and Tables

**Table 1:** Characteristics of the three most common single nucleotide polymorphisms (SNPs) of the *PADI4* gene.

	SNP 163	SNP 245	SNP 335
Reference	rs11203366	rs11203367	rs874881
bp change	A > G (missense)	C > T (missense)	C > G (missense)
MAF (Ensembl)	47.5% (G)	46.7% (T)	47.8% (G)
Amino acid mutation	Gly55Ser	Val82Ala	Gly112Ala

**Table 2:** Primer sequences used to detect SNPs of the *PADI4* gene and resulting amplicon lengths.

SNP	Primer	Primer Sequence (5′—3′)	Amplicons			Primer Location
			Flanking	Allele Specific		
			Length (bp)	Length (bp)	Specificity	
**163A>G**	**IFP**	**GTCGTGGATATTGCCCCCG**	-	} 128	G-allele	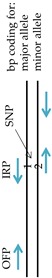
	**ORP**	TCTGGTCGCCTGTGCTACCA	} 251			
	**OFP**	TGCTGGGAGAGCCATGGC		} 166	A-allele	
	**IRP**	GGATTTCTTCTTGGCTGGAGGTCT	-			
**245C>T**	**IFP**	GGTGACCCTGACGATGAACGT	-	} 131	T-allele	
	**ORP**	GTGGATCACGGCAGGACAGA	} 260			
	**OFP**	CTGCCCCTGAGGACTGCAC		} 170	C-allele	
	**IRP**	GCCTGTGCTACCACTGGACG	-			
**335C>G**	**IFP**	CAAAGCTCTACTCTACCTCACGGG	-	} 183	G-allele	
	**ORP**	ACTCCCAGATGTCTGACTGGCT	} 387			
	**OFP**	GCTTTCCCTCCATTCCCATC		} 248	C-allele	
	**IRP**	TGGTTGTCACTTACCCAGCG	-			

IFP: Inner forward primer, ORP: Outer reverse primer, OFP: Outer forward primer, IRP: Inner reverse primer.

**Table 3:** Reaction mixture for the SNP characterization with tetra-primer amplification refractory mutation system PCR (ARMS-PCR) contains in 10 µL.

SNP	163A > G	245C > T	335C > G
**Genomic DNA**	150 ng	50 ng	100 ng
**OFP**	1 pmol	1 pmol	1 pmol
**ORP**	1.5 pmol	1 pmol	1 pmol
**IFP**	4 pmol	5 pmol	1 pmol
**IRP**	2 pmol	2.5 pmol	2 pmol
**T_a_**	66 °C	65 °C	62 °C

**Table 4:** Time and cost to analyze 10 samples (three SNPs in duplicates + controls) with the different SNP detection methods, including method accuracy.

	DNA Isolation	HRM-PCR	PCR-RFLP	ARMS-PCR	One-Step ARMS-PCR
**Handling time (min)**	20	90	120	60	40
**Pause time (min)**	25	330	245–540	150	180
**Total time (min)**	45	420	365–660	210	220
**Material costs (€)**	1.81	31.45	>120	14.51	31.49
Personnel * costs (€)	10	45	60	30	20
**Total costs (€)**	11.81	76.45	>180	44.51	51.49
**Accuracy ° (%)**	-	76.7	100	100	93.3

* technical assistant (30 €/h); ° %-comparison of the obtained results between three different investigators.

## Supplementary Materials

The following are available online at https://www.mdpi.com/2409-9279/2/3/63/s1, Figure S1: Restriction enzymes and associated restriction pattern identified to be suitable for the detection of SNPs in the *PADI4* gene.
