# Supplementary material for: One-Step ARMS-PCR for the Detection of SNPs—Using the Example of the PADI4 Gene
Source: Methods Protoc. 2019 Jul 25;2(3):63. doi: 10.3390/mps2030063 (PMC6789486; doi:10.3390/mps2030063)
Supplement: Supplementary file 1 [file mps-02-00063-s001.pdf]

methods and protocols

MDPI

unique restriction sites within the SNP regions

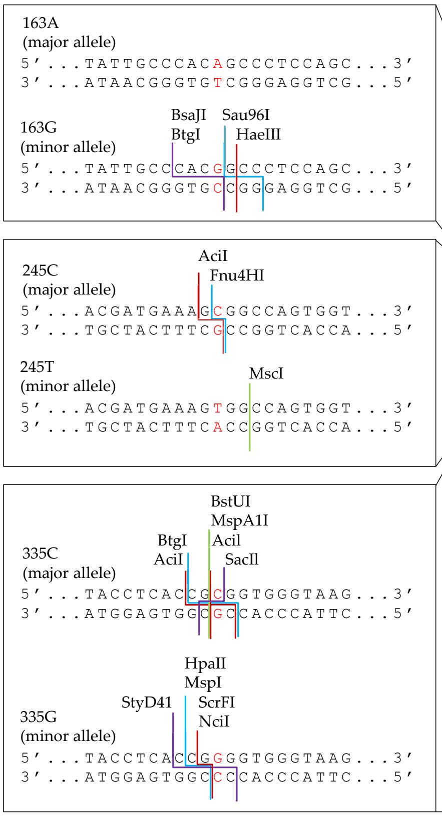

SNP	restriction enzyme	expected band profile [bp]		
		homozygous (minor allele)	heterozygous (both alleles)	homozygous (major allele)
163A>G	BsaJI	103 / 57 / 47 / 23 / ≤12	103 / 70 / 57 / 47 / 23 / ≤12	103 / 70 / 57 / ≤ 12
	BtgI	127 / 112 / 12	239 / 127 / 112 / 12	239 / 12
	Sau96I	123 / 61 / 40 / ≤20	163 / 123 / 61 / 40 / ≤20	163 / 61 / ≤20
	HaeIII	123 / 43 / 40 / 24 / 21	163 / 123 / 43 / 40 / 24 / 21	163 / 43 / 24 / 21
245C>T	MscI	153 / 107	260 / 153 / 107	260
	AciI	260	260 / 150 / 110	150 / 110
	Fnu4HI	228 / 32	228 / 151 / 77 / 32	151 / 77 / 32
335C>G	StyD41	225 / 76 / 66 / 20	245 / 225 / 76 / 66 / 20	245 / 76 / 66
	HpaII	161 / 134 / 92	295 / 161 / 134 / 92	295 / 92
	MspI	161 / 134 / 92	295 / 161 / 134 / 92	295 / 92
	ScrFI	227 / 76 / 64 / 20	247 / 227 / 76 / 64 / 20	247 / 76 / 64
	NciI	227 / 160	387 / 227 / 160	387
	AciI	387	387 / 226 / 159 / 2	226 / 159 / 2
	BtgI	387	387 / 226 / 161	226 / 161
	BstUI	387	387 / 228 / 159	228 / 159
	MspA1I	387	387 / 228 / 159	228 / 159
	SacII	387	387 / 229 / 158	229 / 158

Figure S1: Restriction enzymes and associated restriction pattern identified to be suitable for the detection of SNPs in the *PADI4* gene. Restriction sites identified to be unique for the specific alleles are marked in the region of the SNPs (163A>G, 245C>T, 335C>G). Restriction enzymes and expected restriction patterns were identified with the NEBcutter: http://www.labtools.us/nebcutter-v2-0/ and SNP cutter http://bioinfo.bsd.uchicago.edu/SNP_cutter [Zhang et al. 2005].

Reference: Zhang, R.; Zhu, Z.; Zhu, H.; Nguyen, T.; Yao, F.; Xia, K.; Liang, D.; Liu, C. Snp cutter: A comprehensive tool for snp pcr-rflp assay design. *Nucleic Acids Res* **2005**, *33*, W489-492.
